# Refractory Hypokalemia in the Context of Bilateral Adrenal Hyperplasia Leading to Diagnostic Reassessment and Curative Surgery

**DOI:** 10.7759/cureus.83279

**Published:** 2025-04-30

**Authors:** Mohamed Alaa Sobhi, Larbi Hamedoun, Abdessamad Elbahri, Mohammed Alami, Ahmed Ameur

**Affiliations:** 1 Urology, Mohammed V Military Hospital, Rabat, MAR

**Keywords:** adenoma, adrenalectomy, adrenal mass, bilateral adrenal hyperplasia, refractory hypokalemia

## Abstract

Primary hyperaldosteronism is a common cause of secondary hypertension and is often underdiagnosed. While bilateral adrenal hyperplasia is the most frequent form, the condition may evolve, and a unilateral aldosterone-producing adenoma can emerge, especially in cases presenting with worsening biochemical abnormalities. We report a case of a 60-year-old male patient with a history of bilateral adrenal hyperplasia who presented with life-threatening, asymptomatic hypokalemia (1.41 mmol/L), refractory to intravenous potassium supplementation. Electrocardiography revealed flattened T waves and prominent U waves. A contrast-enhanced CT scan showed a 22 × 19 mm right adrenal mass consistent with a benign cortical adenoma. Due to the urgency of the presentation, adrenal vein sampling was not performed. The patient underwent a laparoscopic right adrenalectomy, which resulted in rapid normalization of serum potassium and improved blood pressure control. Histopathological analysis confirmed an aldosterone-producing adrenal cortical adenoma with background nodular hyperplasia. This case highlights the potential progression of primary hyperaldosteronism, the importance of reassessing adrenal pathology in the presence of severe hypokalemia, and the effectiveness of surgical intervention in resolving both biochemical and hemodynamic disturbances.

## Introduction

Primary hyperaldosteronism (PHA), first described by Jerome Conn in 1955, is a major cause of secondary hypertension, accounting for up to 10% of all hypertensive patients and as high as 20% in those with resistant hypertension [1,2]. PHA is characterized by autonomous overproduction of aldosterone, leading to sodium retention, potassium wasting, and suppression of plasma renin activity. The condition is independent of the renin-angiotensin system and is now increasingly recognized due to more widespread screening in hypertensive populations [3].

The two most frequent subtypes of PHA are bilateral adrenal hyperplasia (approximately 60%) and aldosterone-producing adenoma (around 35%), as confirmed in recent cohort studies and clinical reviews [4,5].

Although hypokalemia is not always present, it remains a hallmark clue, especially in more severe or long-standing diseases. Life-threatening hypokalemia (defined as <2.5 mmol/L) is rare but signifies urgent aldosterone-driven renal potassium loss and potential cardiovascular complications, including ventricular arrhythmias [6].

Accurately distinguishing unilateral from bilateral aldosterone secretion is essential, as it directly determines the therapeutic approach: surgical resection in unilateral APA versus mineralocorticoid receptor antagonism in bilateral hyperplasia. This distinction is typically made via adrenal vein sampling (AVS), though imaging may be relied upon in urgent clinical contexts [7].

This case illustrates a unique scenario in which a 60-year-old man with a long-standing diagnosis of hypertension and presumed bilateral adrenal hyperplasia (BAH) developed severe, treatment-resistant hypokalemia (1.41 mmol/L) during a routine preoperative assessment. The previously stable clinical course masked the emergence of a dominant aldosterone-producing adenoma, ultimately revealed through refractory electrolyte imbalance. This case highlights the evolving nature of adrenal pathology and underscores the need for re-evaluation when biochemical control unexpectedly deteriorates.

## Case presentation

A 60-year-old man with a long-standing history of hypertension, diagnosed over a decade ago and subsequently attributed to bilateral adrenal hyperplasia (BAH), was referred to the emergency department following the incidental discovery of profound hypokalemia during a routine laboratory evaluation. The serum potassium was measured at 1.41 mmol/L. Notably, the patient was asymptomatic upon presentation, refuting the presence of palpitations, muscle cramps, exhaustion, paresthesias, or any other neuromuscular symptoms commonly linked to severe hypokalemia. His persistent antihypertensive prescription included a calcium channel blocker and spironolactone, the latter initiated eight years earlier following the diagnosis of BAH, with both medications having previously maintained adequate blood pressure control.

Upon arrival, vital indicators were within normal parameters. Blood pressure was recorded at 130/67 mmHg, heart rate at 64 beats per minute, and body temperature at 36.8°C. The physical examination revealed no abnormalities, with no indications of volume depletion, neuromuscular irritability, or heart decompensation.

Initial laboratory workup confirmed the severity of the hypokalemia (Table 1).

**Table 1:** Patient's laboratory values

Laboratory test	Patient's result	Reference range
Potassium (K⁺)	1.41 mmol/L	3.5–5.0 mmol/L
Sodium (Na⁺)	146 mmol/L	135–145 mmol/L
Magnesium (Mg²⁺)	2.24 mg/dL	1.6–2.6 mg/dL
Corrected calcium (Ca²⁺)	88 mg/L	86–100 mg/L
Bicarbonate (HCO₃⁻)	28 mmol/L	22–28 mmol/L
Hemoglobin (Hb)	15 g/dL	13.5–17.5 g/dL
Serum creatinine	0.8 mg/dL	0.6–1.2 mg/dL

**Figure 1:**
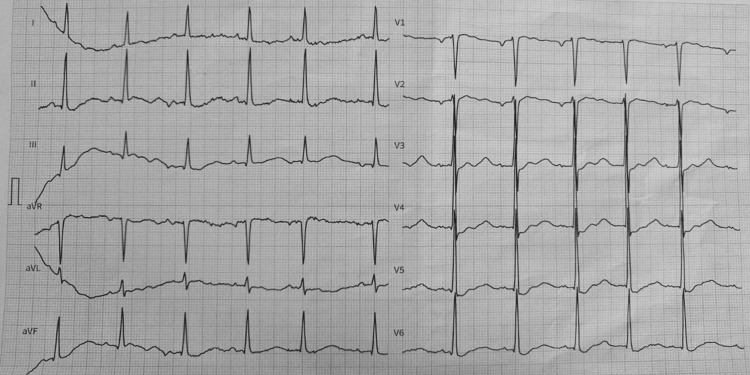
Patient’s electrocardiogram at the time of admission The 12-lead ECG shows sinus rhythm with a heart rate of approximately 64 bpm. There are notable flattened T waves, prominent U waves, and mild ST segment depression, particularly visible in leads V2–V4. These findings are characteristic of severe hypokalemia, consistent with the patient's critically low serum potassium level of 1.41 mmol/L. No ventricular arrhythmias or QT prolongation were observed at the time of recording, but the electrocardiographic profile underscores the risk of malignant arrhythmias without urgent correction.

An electrocardiogram (ECG) revealed sinus rhythm, with flattened T waves and prominent U waves, which are hallmark findings of hypokalemia and indicative of increased risk for ventricular arrhythmias (Figure 1). Given the life-threatening nature of these findings, immediate medical intervention was initiated.

The patient was admitted to the intensive care unit (ICU) for close monitoring and rectification of the electrolyte imbalance. Central venous access was obtained, and the patient commenced vigorous intravenous potassium chloride replacement therapy at a rate of 20 mmol/h, delivered centrally to reduce discomfort and facilitate larger infusion rates. Despite three successive potassium infusions amounting to almost 120 mmol, serum potassium levels remained consistently low, fluctuating between 1.5 and 2.4 mmol/L and never exceeding 2.5 mmol/L. This resistance to replacement medication strongly indicated persistent renal potassium loss, perhaps due to uncontrolled aldosterone output.

Given the patient's established history of BAH, the unanticipated severity and persistence of the hypokalemia suggested the potential presence of a superimposed or newly dominant aldosterone-producing adenoma (APA). A reassessment of adrenal pathology was considered essential.

**Figure 2:**
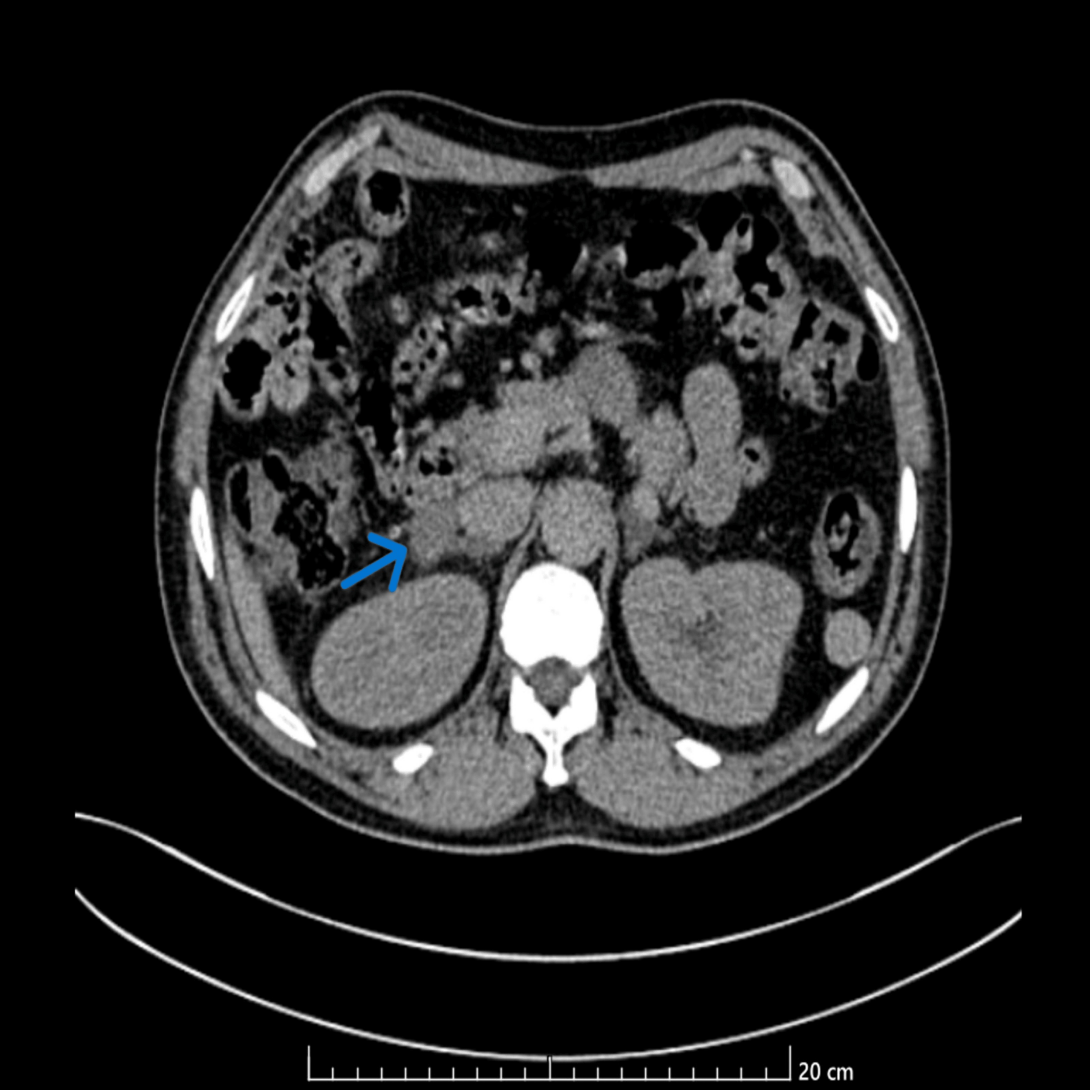
: Axial contrast-enhanced abdominal CT scan demonstrating a right adrenal mass (blue arrow)

To review the adrenal glands, a contrast-enhanced abdomen computed tomography (CT) scan was conducted. The scan found a right adrenal mass measuring 22 x 19 mm on the lateral branch of the adrenal gland. The lesion showed a pre-contrast density of 12 Hounsfield Units (HU) and a post-contrast increase of 36 HU, with a washout above 60% at 90 seconds, matching the imaging characteristics of a benign cortical adenoma (Figure 2).

These results strongly pointed to a functional adrenal adenoma and suggested that this lesion, rather than diffuse bilateral hyperplasia, was the main cause of aldosterone excess.

Adrenal vein sampling (AVS) was not sought given the clinical urgency; it is the gold standard for lateralizing primary aldosteronism. The clinical setting and imaging results were considered instead to be adequate to go on with surgical intervention.

Given the ongoing, life-threatening hypokalemia, lack of reaction to conservative treatment, and radiographic proof of a dominant adrenal lesion, a multidisciplinary conversation with the urology and endocrinology departments guided the choice to undergo a right laparoscopic adrenalectomy.

**Figure 3:**
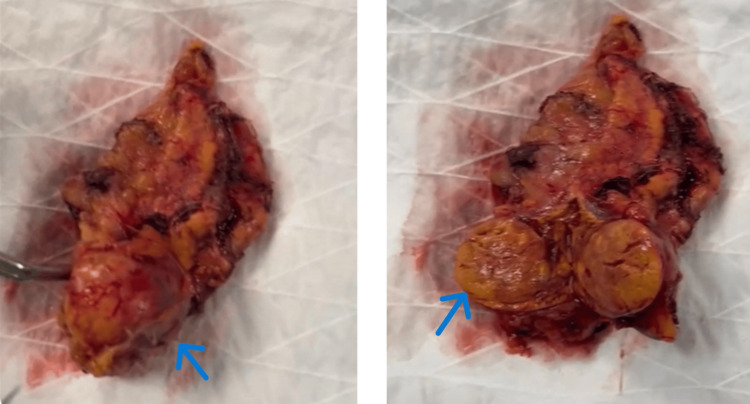
Gross anatomical specimen of the excised right adrenal gland following laparoscopic adrenalectomy On the left, the gland appears intact with a visible well-circumscribed adrenal mass on the inferior pole (blue arrow). On the right, a cross-sectional view shows a well-encapsulated nodule, with a yellow-tan cut surface and minimal hemorrhage, consistent with cortical adenoma (blue arrow).

Preoperative stabilization kept the patient in the ICU, continuous potassium infusion was kept to progressively raise serum potassium levels. on the morning before surgery, the serum potassium had risen to 2.8 mmol/L, deemed acceptable for safe anesthesia and operational intervention,

The procedure was carried out under general anesthesia using a laparoscopic transperitoneal approach. The pneumoperitoneum was created using carbon dioxide after the patient was positioned in a left lateral decubitus posture. Three trocars were inserted: two for working tools and one for the camera. The hepatic flexure of the colon was mobilized and the right lobe of the liver was retracted superiorly to reveal the retroperitoneum. To find the right adrenal vein, dissection started at the lateral side of the inferior vena cava; it was clipped and split with endoscopic vascular clips. With great care to preserve surrounding tissues, the adrenal gland and tumor mass were surgically dissected en bloc. Consistent with imaging features of a benign adenoma, the adrenal mass was soft, encapsulated, and clearly delineated. There were no intraoperative problems and hemostasis was easily attained. An endoscopic bag was used to collect the specimen (Figure 3). With little blood loss (<50 mL), the total operation time was around 95 minutes. Postoperative monitoring in the ICU was passed to the patient, who was successfully extubated.

There was no eventful immediate postoperative course. The serum potassium normalized to 4.2 mmol/L after 72 hours without further supplementation. Significant reductions in blood pressure as well as steady tapering of antihypertensive treatment. By postoperative day three, spironolactone was completely stopped.

The histopathology confirmed the diagnosis of an aldosterone-producing adrenal cortical adenoma, with nodular hyperplasia. There was no evidence of malignancy.

The patient was discharged home on postoperative day five, with instructions for endocrine follow-up and gradual reduction of his remaining antihypertensive medications.

## Discussion

This case underscores the clinical complexity and dynamic nature of primary hyperaldosteronism (PHA), particularly in patients with an initial diagnosis of bilateral adrenal hyperplasia (BAH). While BAH typically warrants lifelong medical therapy with mineralocorticoid receptor antagonists, the emergence of refractory hypokalemia in a previously stable patient necessitates diagnostic reconsideration and highlights the potential for an evolving or superimposed aldosterone-producing adenoma (APA).

Hypokalemia is a classic, though not universal, feature of PHA. When present, it indicates the severity of aldosterone-induced renal potassium loss induced by aldosterone. The patient's potassium level of 1.41 mmol/L was critically low and unresponsive to vigorous replacement, suggesting the possibility of persistent endogenous mineralocorticoid excess. This phenomenon has gained recognition in instances of functioning adrenal adenomas or predominant nodules inside a hyperplastic gland [8].

Recent literature supports the view that refractory or severe hypokalemia in PHA is associated with a higher likelihood of surgical disease and worse cardiovascular outcomes [3,6]. A recent retrospective review of adrenal incidentalomas found that potassium levels <2.5 mmol/L were strongly associated with curable forms of PHA, reinforcing the need for early imaging and consideration of surgical intervention [3].

Although BAH is regarded as a diffuse condition, recent studies indicate that nodular transformation or the emergence of a dominant functional adenoma may develop over time. Genetic and somatic mutations (e.g., KCNJ5, CACNA1D) are recognized as catalysts for clonal aldosterone production, especially in cases of previous bilateral hyperplasia [9]. Consequently, the diagnostic designation of BAH should not inhibit re-assessment in individuals exhibiting biochemical decline or unusual advancement. Imaging in this patient demonstrated a well-defined right adrenal mass exhibiting low pre-contrast density, substantial contrast washout, and radiological characteristics indicative of a benign adenoma. While adrenal vein sampling (AVS) remains the gold standard for lateralizing aldosterone secretion, in urgent clinical settings or where AVS is inconclusive or unavailable, CT-based anatomical localization paired with strong clinical and biochemical evidence may be sufficient to guide surgical decision-making [10,11].

The decision to proceed with laparoscopic adrenalectomy was validated by imaging and the ineffectiveness of pharmacological therapy. Laparoscopic transperitoneal adrenalectomy is the preferred method due to its low morbidity, rapid recovery, and improved visualization. The surgical resolution of hypokalemia and improvement of blood pressure in this patient correspond with current studies demonstrating that surgery is curative in over 90% of APA patients, particularly when performed expeditiously [12].

Patients with chronic PHA may exhibit renal tubular adaptations or cardiovascular remodeling that persist after surgery. The normalization of potassium within 72 hours in this patient is diagnostically confirmatory and prognostically beneficial [6]. Postoperative hypoaldosteronism may arise in specific cases, particularly in patients with reduced contralateral gland functionality. While not apparent in the present case, long-term monitoring, including vigilance for hyperkalemia and hypotension, is essential [12].

This case reinforces the necessity of ongoing surveillance in patients diagnosed with BAH, even those long considered biochemically stable. Clinicians should maintain a low threshold for re-investigation in the face of unexplained or refractory hypokalemia. Early recognition of evolving or atypical PHA phenotypes enables timely surgical intervention, with the potential for cure and prevention of long-term end-organ damage.

## Conclusions

This case illustrates how a previously diagnosed bilateral adrenal hyperplasia can mask a surgically correctable aldosterone-producing adenoma. The presence of severe, refractory hypokalemia should prompt reassessment of adrenal function and morphology. Timely diagnosis and adrenalectomy led to rapid correction of electrolyte imbalance and improved blood pressure control, highlighting the importance of re-evaluating treatment-resistant cases of primary aldosteronism.
